# Isolated Cyclic Loading During Adolescence Improves Tibial Bone Microstructure and Strength at Adulthood

**DOI:** 10.1002/jbm4.10349

**Published:** 2020-03-11

**Authors:** Tanvir Mustafy, Irène Londono, Florina Moldovan, Isabelle Villemure

**Affiliations:** ^1^ Department of Mechanical Engineering École Polytechnique of Montréal Montréal Québec Canada; ^2^ Department of Pediatrics Sainte‐Justine University Hospital Center Montréal Québec Canada; ^3^ Department of Stomatology, Faculty of Dentistry Université de Montréal Montréal Québec Canada

**Keywords:** BIOMECHANICS, BONE ADAPTATION, DETRAINING, GROWTH AND DEVELOPMENT, MECHANICAL LOADING

## Abstract

Bone is a unique living tissue, which responds to the mechanical stimuli regularly imposed on it. Adolescence facilitates a favorable condition for the skeleton that enables the exercise to positively influence bone architecture and overall strength. However, it is still dubious for how long the skeletal benefits gained in adolescence is preserved at adulthood. The current study aims to use a rat model to investigate the effects of *in vivo* low‐ (LI), medium‐ (MI), and high‐ (HI) intensity cyclic loadings applied during puberty on longitudinal bone development, morphometry, and biomechanics during adolescence as well as at adulthood. Forty‐two young (4‐week‐old) male rats were randomized into control, sham, LI, MI, and HI groups. After a 5 day/week for 8 weeks cyclic loading regime applied on the right tibia, loaded rats underwent a subsequent 41‐week, normal cage activity period. Right tibias were removed at 52 weeks of age, and a comprehensive assessment was performed using μCT, mechanical testing, and finite element analysis. HI and MI groups exhibited reduced body weight and food intake at the end of the loading period compared with shams, but these effects disappeared afterward. HI cyclic loading increased BMD, bone volume fraction, trabecular thickness, trabecular number, and decreased trabecular spacing after loading. All loading‐induced benefits, except BMD, persisted until the end of the normal cage activity period. Moreover, HI loading induced enhanced bone area, periosteal perimeter, and moment of inertia, which remained up to the 52nd week. After the normal cage activity at adulthood, the HI group showed increased ultimate force and stress, stiffness, postyield displacement and energy, and toughness compared with the sham group. Overall, our findings suggest that even though both trabecular and cortical bone drifted through age‐related changes during aging, HI cyclic loading performed during adolescence can render lifelong benefits in bone microstructure and biomechanics. © 2020 The Authors. *JBMR Plus* published by Wiley Periodicals, Inc. on behalf of American Society for Bone and Mineral Research.

## Introduction

Bone is a unique living tissue that responds to mechanical stimuli regularly imposed on it.^1^, ^2^ Mechanical forces are considered beneficial to the skeleton at an early age for promoting healthy bone growth by increasing bone mass and mineral content through a bone modeling process.^2^, ^3^ This concept has been established for ages by rigorous theories and hypotheses,^4^ and relationships have been formulated to correlate bone geometric and structural developments with respect to undergoing bone mechanical stresses.^5^ During the adolescent period, rapidly growing bones react sensitively to induced mechanical loadings.^6^ Adolescence offers a favorable condition for the skeletal response to mechanical loadings, where impact exercise positively influences bone architecture and overall strength.^7^, ^8^ Indeed, both positive and negative influences in skeletal development and bone geometry were observed—caused by daily mechanical loadings from physical activities.^9^ Mechanical loadings can be induced by compression/tension, bending, shear, or torsion, depending on the type of physical activities.^10^ However, physical activities producing higher ground reaction forces (ie, impact exercise such as running, jumping, hiking, etc.) were shown to be more effective for strengthening bone microstructure.^11^

For loading‐based bone modification induced during adolescence, it has been hypothesized that it could have an impact later in life if the effect persists at adulthood.^12^, ^13^ However, it is still not clear for how long skeletal benefits gained during adolescence could be preserved at adulthood. Animal and clinical studies have been conducted to investigate the effects of pubertal loading impact on bony structures. Clinical studies have reported that different forms of physical activities performed during adolescence resulted in 10% to 15% greater bone mass in the participating children compared with the nonparticipating ones.^14^, ^15^, ^16^ A study of baseball players reported that the effects of ball‐throwing persisted throughout life in the form of additional bone strength.^3^ However, few studies have concluded that pubertal loading could induce a bone mass increase, which prevailed for a short period but diminished over time and disappeared in adulthood.^17^, ^18^ Similar to clinical investigations, animal studies have also shown contradictory results. In different studies,^2^, ^19^, ^20^ researchers observed that bone structural changes during puberty caused by induced loading tend to last long into adulthood. However, contradictory findings showed the absence of skeletal benefits and even bone loss phenomena at adulthood.^19^, ^21^ This discrepancy could be associated with animal ages or genders, study design, and exercise protocols. Most of the experimental studies started the exercise and/or loading regime at the middle or end of the adolescent period (approximately 1.5 to 2 months old).^22^, ^23^, ^24^, ^25^, ^26^ So, these studies lack the data for the entire adolescent period, which is considered one of the most crucial periods for bone development in rats.^7^, ^8^ Moreover, these studies investigated bone mass and mineral content at limited time points rather than looking at the longitudinal data, which would give a better understanding about the bone modeling dynamics and the temporal nature of bone response to applied mechanical stimuli. Two studies investigating adolescence exercise effects on rat limb found contrary results regarding persisting skeletal benefits gained during the pubertal period. One study reported decreasing positive effects in the femoral midshaft and femoral neck in 47‐week‐old rats^27^ whereas another reported enhanced bone strength in 97‐week‐old rat tibias.^2^ Moreover, none of these studies assessed these changes longitudinally on both trabecular and cortical bone microstructures.

Hence, it is still not clear whether a controlled isolated cyclic loading regime during puberty longitudinally influences bone quantity, quality, and mechanics, and if/how long these changes persist into adulthood. The current study aimed to investigate the effects of in vivo cyclic loadings (low‐, medium‐, and high‐intensity compression) applied during puberty on longitudinal bone development, morphometry, and biomechanics at the end of adolescence as well as during adulthood using a rat tibial model. Rat tibias were scanned from 4 to 52 weeks of age to assess trabecular and cortical bone changes to loading using in vivo μCT. At euthanization (52 weeks old), bone biomechanical properties were extracted from three‐point bending tests; strains were also investigated based on simulations of axial compression using voxel‐based finite element models.

## Materials and Methods

### Animals

Forty‐two male Sprague–Dawley rats (Charles River Laboratories, Montreal, Canada) were received at approximately 3 weeks of age. Rats were housed two per cage with *ad libitum* access to food and water, and were kept at 25°C with a 12‐hour light/dark cycle. All animal experiments were carried out according to the policies of the Canadian Council on Animal Care (CCAC), and procedures were approved by the Institutional Animal Care Committee at Sainte‐Justine University Hospital, Montreal, Canada. After 1 week of acclimatization to the diet and new environment, rats were randomly divided into five groups: control, sham, low intensity (LI), medium intensity (MI), and high intensity (HI). Control and sham groups consisted of 6 animals; each impact group consisted of 10 animals. Both body weight (BW) and food intake (FI) were monitored weekly during the adolescent loading period (until the end of the 11th week) and monthly during the normal cage activity period (12th to 52nd week) to monitor overall health.

### Isolated tibial cyclic loading

**Figure 1:**
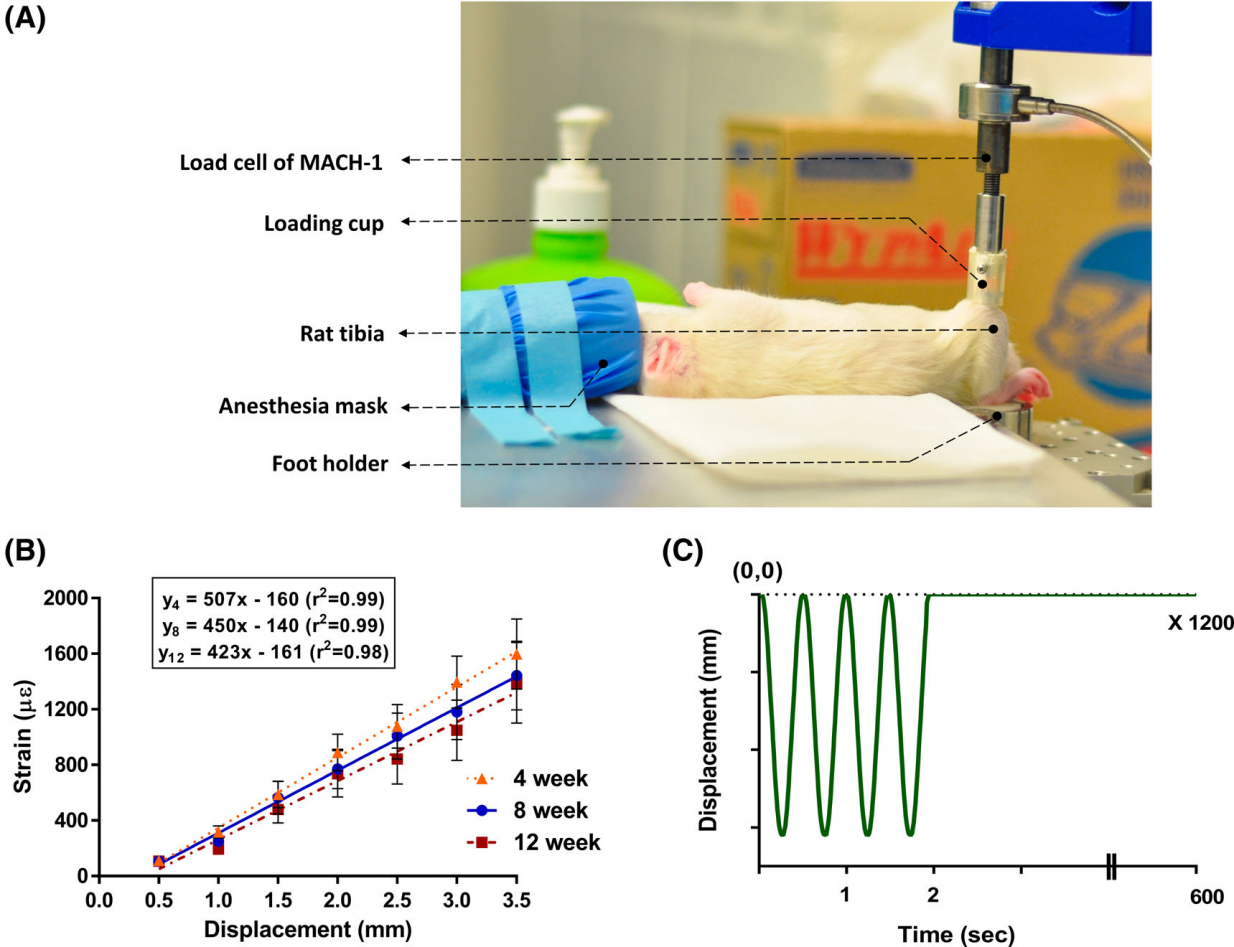
(*A*) *in vivo* cyclic loading of the right tibia of an 8‐week‐old rat. (*B*) Strain gauge calibration curves at the medioproximal tibial surface for 4‐, 8‐, and 12‐week‐old rats. Error bars represent SDs (*n* = 6 rats/age group). (*C*) Representative *in vivo* cyclic loading profile including 1200 repetitions over approximately 10 min/day. Peak‐to‐peak displacements were chosen based on the strain gauge calibration curves previously obtained for the three age groups.

**Figure 2:**
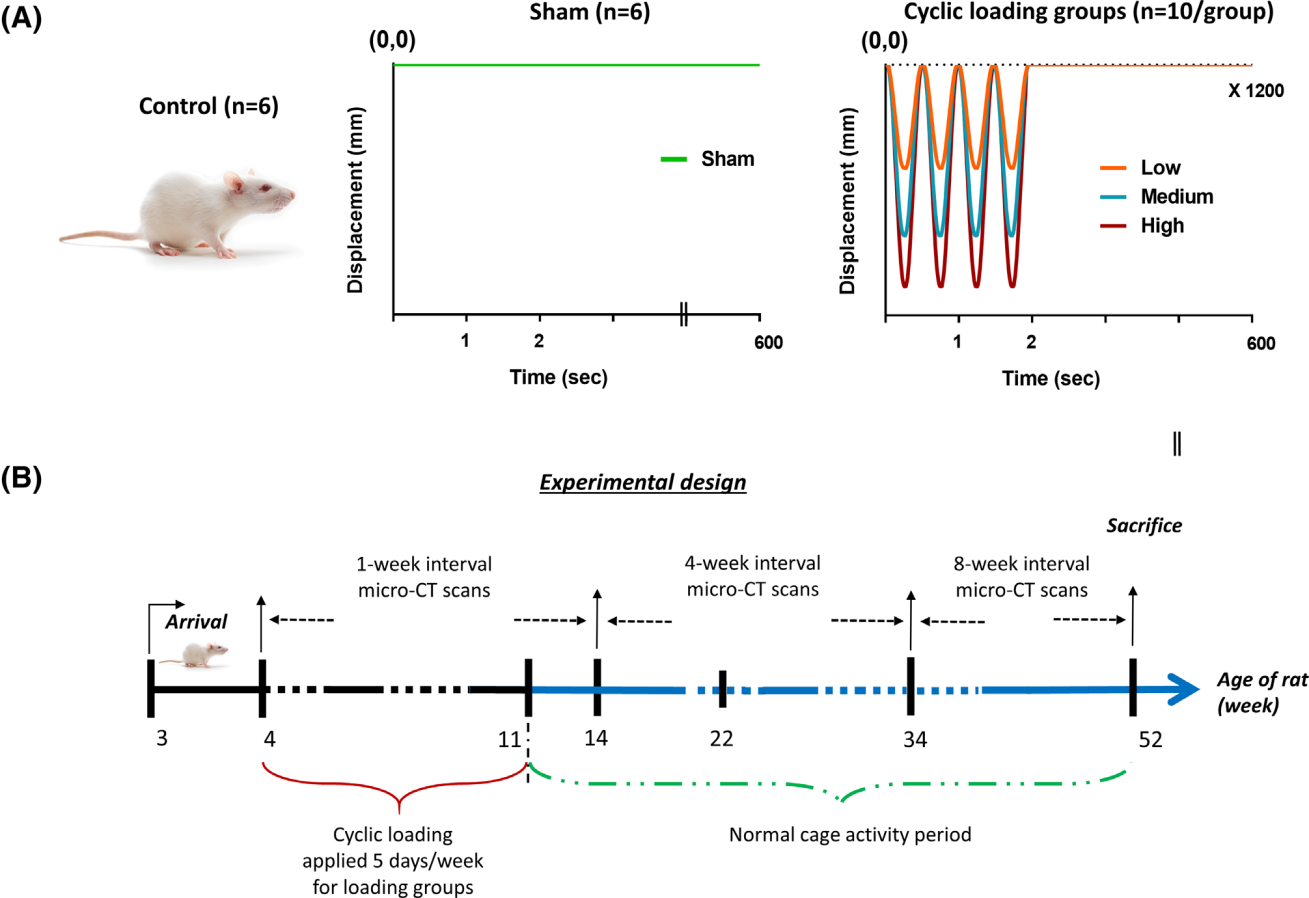
(*A*) Five rat groups (*n* = 42 total) were used: control (C; *n* = 6), sham (S; *n* = 6), low intensity (LI; *n* = 10), medium intensity (MI; *n* = 10), and high intensity (HI; *n* = 10). The right tibia of each rat from LI, MI, and HI groups were loaded using the waveform, respectively, triggering 450, 850, and 1250 με tensile strain at the medio‐proximal tibial surface from 4 to 11 weeks of age, corresponding to rat adolescence. (*B*) Cyclic loadings were applied 5 days/week from 4 to 11 weeks of age. Rats were detrained from the 11th to 52^nd^ week. At the end of the experiment (52 weeks old), rats were euthanized, both structural and estimated tissue‐level mechanical properties were obtained. Right tibias were scanned during the entire experimental period, at different time intervals, for acquiring in vivo bone microstructural parameters.

Tibial cyclic loading for LI, MI, and HI groups began at 4 weeks of age using a custom‐built cyclic loading device (Fig. 1 *A*). Rats were anesthetized (2% isoflurane, 1.0 L/min O_2_) during cyclic impact loading, which was controlled using a Mach‐1 V500C (Biomomentum Inc., Montreal, Canada) to apply a 2‐Hz haversine waveform for 1200 cycles/day, 5 days/week for 8 weeks.^28^ A compressive preload of 0.5 N was applied to keep the tibia in a steady position. The cyclic loading was characterized by symmetric active loading/unloading with a 0.10 s of rest period between load cycles (Fig. 1 *C*).^29^, ^30^, ^31^, ^32^ These three loading conditions varied not only in terms of displacement (strain) magnitude, but also in terms of the acceleration applied during loading and unloading conditions. The relationship between applied displacements and peak strains at the medio‐proximal surface of the right tibia was established in preliminary compression‐strain calibration experiments with 18 rats 4, 8, and 12 weeks old (*n* = 6/age group; Fig. 1 *B*). The axial displacement values generating 450, 850, and 1250 με tensile strain at the medio proximal tibial surface were used for LI, MI, and HI groups, respectively (Fig. 2 *A*). These strain magnitudes correspond to peak tensile strain values in the human tibia during unrestricted walking (450 με), zig‐zag uphill running (850 με), and vertical jumping (1250 με) conditions.^33^, ^34^, ^35^, ^36^ Also, the lowest selected peak strain (450 με) has been reported to be sufficient to induce bone adaptation.^37^, ^38^ Linear interpolation was applied to extract displacement values for the weeks in‐between the chosen calibration ages. Similar experimental manipulations were applied to the sham rats without any axial loading. Controls were kept in the cage without any manipulation. For all rats, normal cage activity was allowed between loading sessions.

### μCT

#### μ‐CT scanning regime

**Figure 3:**
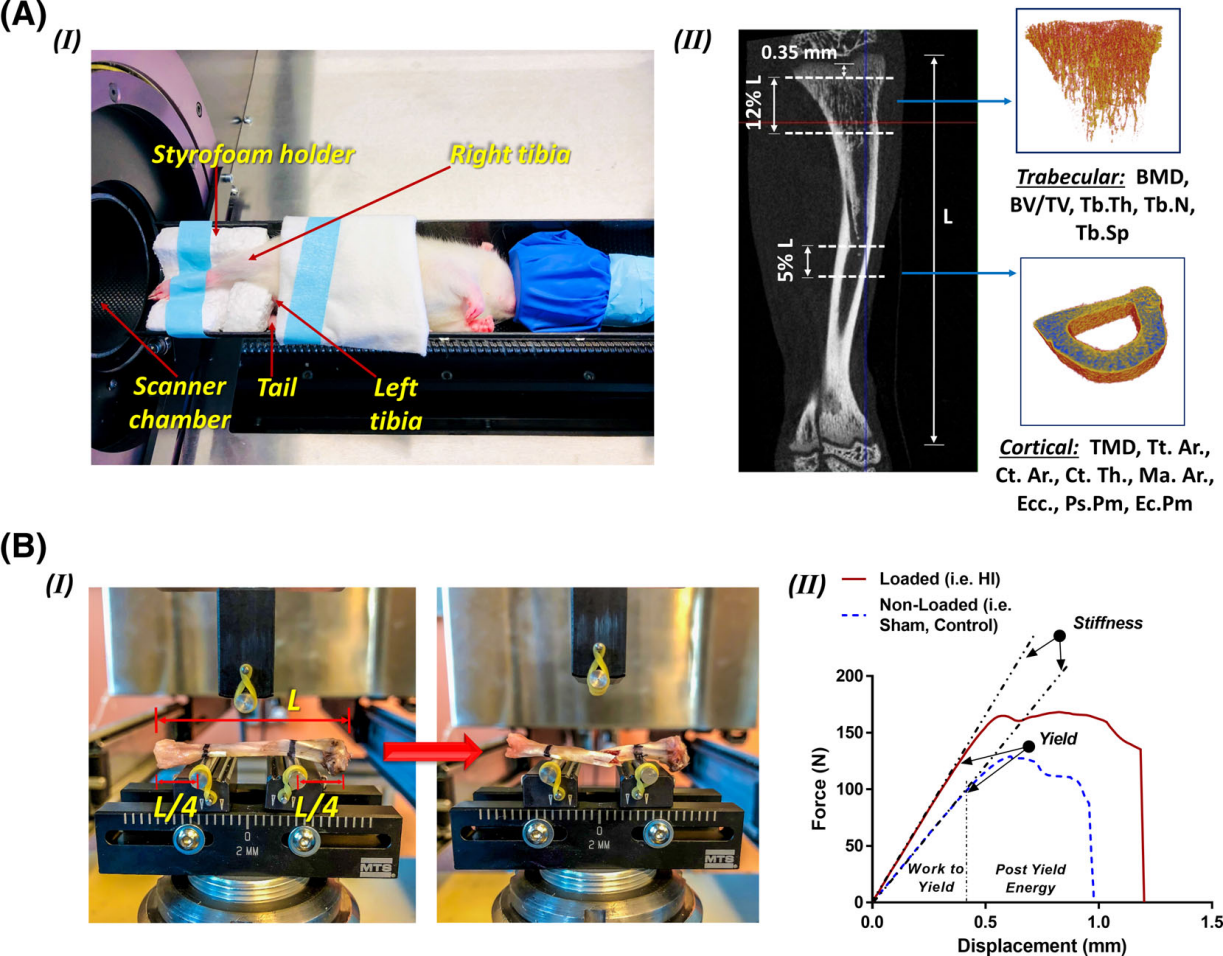
(*A*) (I) Rat positioning for the *in vivo* μCT scanning. Although anesthetized, the rat was placed sideways securing the right tibia into a Styrofoam holder and firmly held with medical adhesive tape. The left tibia was folded toward the animal's head and placed alongside with the tail. (II) representative longitudinal section of a rat tibial CT scan showing the total tibial length (L). The trabecular VOI started at approximately 0.35 mm distal to the growth plate and extended for 12% L. The cortical VOI was fixed at the tibial mid‐diaphysis and equally spanned proximally and distally for 5% L. Using a semiautomatic segmentation algorithm, trabecular and cortical sections were extracted to further evaluate bone morphometric parameters. (*B*) (I) Three‐point bending test experimental set‐up, before and after bone fracture. Half of the total tibial length (L) was set between supports, with the remaining length equally distributed between the external sides of the supports. (II) Representative force versus displacement curves for a high‐intensity tibia and sham tibia after normal cage activity period (52 week old). BV/TV = bone volume fraction; Tb.N = trabecular number; Tb.Th = trabecular thickness; Tb.Sp = trabecular spacing; TMD = tissue mineral density; Tt.Ar = cross‐sectional area inside the periosteal envelope; Ct.Ar = cortical bone area; Ct.Th = cortical thickness; Ps.Pm = periosteum perimeter; Ec.Pm = endocortical perimeter; Ma.Ar = medullary area; Ecc = mean eccentricity.

An in vivo μCT scanner (Skyscan 1176, Skyscan, Aartselaar, Belgium) was used for the longitudinal assessment of the right tibial bone morphology using an isotropic voxel size of 18 μm, 65 kV, 384 μA, 350‐ms exposure time, 0.65‐degree rotation step, no frame averaging, and a 1‐mm Al filter.^39^ The scans were performed at 1‐week intervals from the 4th to 14th week of age, at 4‐week intervals for the next 22 weeks, and at 8‐week intervals for the remaining normal cage activity period of 17 weeks (total 52 week of age; Fig. 2 *B*). The selection of the in vivo μCT radiation doses was made to acquire high‐quality scanned images without interfering with the bone development process. To set a safe radiation dosage level, three sets of radiation doses were investigated for repeated scanning of the right tibia during the adolescent period in a preliminary study.^39^ Three radiation doses (0.83, 1.65, and 2.47 Gy) were selected to produce high‐quality images for bone development investigation purposes. It was observed that the 1.65‐ and 2.4‐Gy radiation doses negatively affected the bone development process, whereas under the 0.83‐Gy radiation dose, bone growth remained unaffected during the scanning period. Accordingly, the 0.83‐Gy radiation dose was selected for this project. Rats were positioned on the carbon‐fiber half‐tube bed of the scanner and kept anesthetized (2% isoflurane, 1.0 L/min O_2_) during the scanning procedure. The right tibia was positioned in a Styrofoam holder of cylindrical shape to ensure its placement in the midline of the scanner (Fig. 3 *A*.I). A phantom calibration was performed on each scanning day using two cylindrical hydroxyapatite phantoms (0.25 and 0.75 g/cm^3^ of calcium hydroxyapatite [CaHA]). Reconstructions of the scanned images were performed using NRecon software (v.1.6.10; Bruker‐μCT, Kontich, Belgium).^39^, ^40^

#### In vivo assessment of trabecular bone morphometry

For each tibia, a trabecular bone volume of interest (VOI) was defined to include the secondary spongiosa in the proximal metaphysis, starting at approximately 0.35 mm distally to the growth plate and extending for 12% of the total tibial length (L) (Fig. 3 *A*.II).^39^, ^41^ The trabecular bone VOI was semiautomatically segmented using an in‐house algorithm to exclude the cortical shell. A global threshold of 65 Gy, corresponding to an equivalent density of 0.413 g/cm^3^ of CaHA, was used for all analyses (CTAn software v.1.13).^39^, ^41^, ^42^ Trabecular bone structural parameters included BMD, bone volume fraction (BV/TV), trabecular number (Tb.N), trabecular thickness (Tb.Th), and trabecular spacing (Tb.Sp).^43^

#### In vivo assessment of cortical bone morphometry

The cortical VOI included the cortical part of the bone and the marrow cavity, centered at the midpoint of the tibial mid‐diaphysis, and equally extended proximally and distally for a total of 5% of the tibial length (L; Fig. 3 *A*.II). A global threshold of 65 Gy was also used for all analyses.^41^, ^42^ Cortical bone structural parameters included tissue mineral density (TMD), cross‐sectional area inside the periosteal envelope (Tt.Ar), cortical bone area (Ct.Ar), cortical thickness (Ct.Th), periosteum perimeter (Ps.Pm), endocortical perimeter (Ec.Pm), medullary area (Ma.Ar), and mean eccentricity (Ecc).^43^ The polar moment of inertia (I_P_, mm^4^) was evaluated as the sum of I_MIN_ and I_MAX_.

### Ex vivo muscle weight measurements

After the last μCT imaging (52nd week), rats were euthanized using CO_2_ asphyxiation, followed by decapitation. The right tibia and femur were then carefully dissected by trained professionals to isolate the gastrocnemius, tibialis anterior, quadriceps femoris, and soleus muscles with a scalpel. A precision electronic scale (Adam PW254 analytical balance, 0.1 mg precision; Adam Equipment, Oxford, CT, USA) was used to evaluate the weight of the isolated muscles (Table 1).

**Table 1:** Muscle Weights (g) for Control, Sham, LI, MI, and HI Groups Evaluated at the End of the Experiment

	Muscle weights (g)			
Groups	Gastrocnemius	Tibialis anterior	Soleus	Quadriceps
Control	4.43 ± 0.39	1.08 ± 0.22	0.26 ± 0.02	4.83 ± 0.18
Sham	4.17 ± 1.19	1.23 ± 0.45	0.24 ± 0.04	5.03 ± 0.28
Low impact (LI)	4.55 ± 0.75	1.02 ± 0.13	0.32 ± 0.04	4.93 ± 0.41
Medium impact (MI)*	4.92 ± 0.60	1.13 ± 0.18	**0.34 ± 0.04** ^**α**^	5.47 ± 0.29
High impact (HI)*	4.82 ± 0.99	1.07 ± 0.22	**0.38 ± 0.07** ^**α**^	**5.64 ± 0.24** ^**α**^

Values are expressed as means ± SDs, *N* = 6/group for control and sham groups; *N* = 9/group for LI and HI; and *N* = 8 for MI. In the group column, * indicates a significant effect (*p* < 0.05) from a two‐way repeated‐measure ANOVA with Tukey's multiple comparisons. When there was a significant effect, Tukey's post hoc pairwise comparisons evaluated whether the sham group was significantly different compared with the other groups. Significant differences are indicated in bold value with “α.”

### Mechanical testing

Right tibias (*n* = 42) from all rat groups were cleaned of soft tissues and tested to failure in three‐point bending under displacement control at 0.15 mm/s using an MTS 793 servo‐hydraulic testing system (MTS Systems Corp., Eden Prairie, MN, USA). A load cell of 100‐kN capacity combined with an MTS three‐point flexural mounting setup was used to rupture the tibias at their midshafts (Fig. 3 *B*.I). Support‐to‐support distance was set at 50% of the total tibial length, while keeping the tibia horizontally centered between the ends (Fig. 3 *B*.I). Force and displacement data were collected every 0.1 s to obtain force versus displacement curves, from which extrinsic biomechanical properties were determined, including the ultimate force (N), yield force (N), work to yield (mJ), work to failure (mJ), and linear stiffness (N/mm). Intrinsic biomechanical properties were also calculated from the cross‐sectional parameters measured from the μCT images at the tibial mid‐diaphysis.^44^ Young's modulus E (GPa) was determined using the moment of inertia, stiffness, and span length.^45^ Yield stress, σ_y_ (MPa) and ultimate stress, σ_ult_ (MPa) were determined using yield and ultimate force, distance from the centroid of the cross‐section to the outermost point on the cross‐section, moment of inertia, and span length.^44^, ^45^ Assuming linear elastic bone material,^45^, ^46^, ^47^ resilience and toughness were determined by the following equations:

(1) $$\text{Resilience}=\frac{\sigma_{y}^{2}}{2E}\;$$

(2) $$\text{Toughness}=0.75*W*\left(\frac{b^{2}}{\mathrm{LI}}\right)\;$$

where σ_*y*_ is the yield stress (MPa), *E* is the Young's modulus (GPa), and *W* is the work to failure (mJ), *b* is the width of the bone cross‐section at the mid‐diaphysis in the anteroposterior direction (mm), *L* is the span length (mm), and *I* is the cross‐sectional moment of inertia (mm^4^).^48^, ^49^

### Finite element analysis

μCT images were used to develop specimen‐specific finite element (FE) models of rat tibia at euthanization (52 weeks old) from all five groups. Average maximum and minimum principal strains were assessed for a proximal and a mid‐diaphysis section of the tibia under a simulated 35‐N compressive force.^49^ This value of the applied compressive force was used in a previous study as a physiologic loading condition not causing any microdamage in the tibia.^49^, ^50^ The investigated VOIs for trabecular and cortical bones were similar to the ones used for the morphometric analyses. μCT images of each tibia were processed using an in‐house MatLab (MathWorks, Natick, MA, USA) mesh generation program to generate a 3D voxel‐based finite element model, where eight‐noded brick elements were used to represent bone voxels.^51^ After a mesh convergence study, the models were created by combining 2 × 2 × 2 pixels in 18‐μm resolution images to yield a single voxel with a side length of 36 μm. Linear elastic, isotropic but nonhomogeneous material properties were assigned to the voxels with a Poisson's ratio of 0.3.^47^, ^52^, ^53^ The elastic modulus was assigned to each voxel based on two calibration steps.^49^ First, a calibration was performed to construct the grayscale‐HU relationship as follows:

(3) HU=18.278*grayscale−1000

Second, a phantom calibration (with CaHA concentrations of 0.25 and 0.75 g/cm^−3^) was performed to construct the following HU‐density relationship:

(4) $$\rho =3.821x10^{-3}*\mathrm{HU}-0.062\;$$

Finally, Young's modulus (*E*) was related to the bone density (ρ) of each voxel using the following equation^54^:

(5) $$E=E_{\max}*{\left(\frac{\rho }{\rho_{\max}}\right)}^{2}\;$$

where *E*_*max*_ = 28.6 GPa, which represented the maximum value of the Young's modulus for the cortical bone structure of 52‐week‐old rat tibias, and ρ_*max*_ = 1.762 *g*/*cm*^−3^, which represented the maximum value of density calculated from all the FE samples used in this study. The compressive force was applied at the proximal end of the tibia in the longitudinal (Z) direction. At the distal end, all nodes were constrained in the X and Y directions to prevent rigid‐body motion. Strains were determined at the element centroids.

### Statistical analysis

Statistical analyses were performed using SPSS Statistics (v. 23; SPSS, Inc., Chicago, IL, USA). An ANOVA test (general linear model) was performed on the BW, FI, and FI relative to BW for the entire experimental period to assess the effects of loading, time, and interaction between BW and FI. To isolate the effects of impact loading, impact groups were compared among themselves and with respect to the sham group. In addition, the control and sham groups were compared to detect any handling and manipulation effects.

Bone structural properties of both trabecular and cortical microstructure from all rat groups were statistically analyzed at 11, 14, 22, 34, and 52 weeks of the experimental period. Biomechanical properties evaluated using three‐point bending tests were also statistically analyzed. Muscle weights as well as strain results from the FE analyses were also statistically compared. In all cases, a two‐way, repeated‐measure ANOVA with Tukey's multiple comparisons was performed to assess the significant group difference and pairwise comparisons. Data are presented as means ± SD. Statistical significance was fixed at *p* < 0.05.

## Results

### Animals

Four animals (9.5%) had to be euthanized at earlier time points before the end of the study period because of dryness in the paws (*n* = 1), anesthesia complications (*n* = 1), and unknown/natural causes (*n* = 2). These animals were excluded from the study. The resulting group sizes for the 11‐, 14‐, 22‐, 34‐, and 52‐week‐old period were then 42, 42, 40 (control/sham, *n* = 6; LI, *n* = 10; MI/HI, *n* = 9), 39 (control/sham, *n* = 6; LI/HI/MI, *n* = 9), and 38 (control/sham, *n* = 6; LI/HI, *n* = 9; MI, *n* = 8), respectively.

### Body weight and food intake

**Figure 4:**
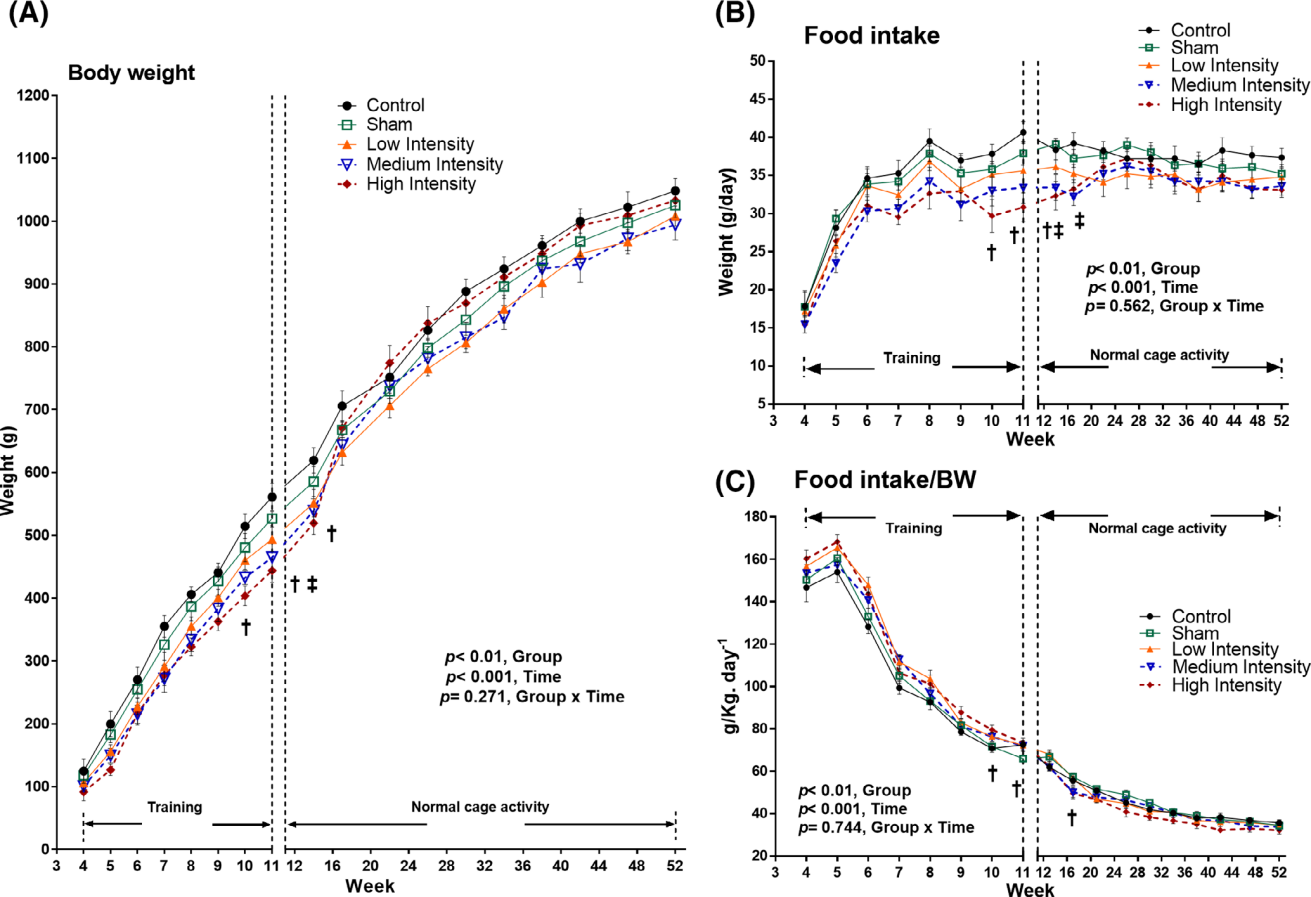
(*A*) Rat body weight (g). ANOVA test (general linear model) was performed to determine time effects, group effects, and their interactions on body weight. (*B*) Absolute daily food intake (g/day). ANOVA test (general linear model) was performed to determine time effects, group effects, and their interactions on food consumption. (*C*) Relative quantity of food intake per unit body weight (g/kg/day^−1^). ANOVA test (general linear model) was performed to determine time effects, group effects, and their interactions on food intake per unit body weight. Graphs are plotted considering the values throughout the experimental period (4 to 52 week of age). Values are presented as means ± SDs; *p* < 0.05 **‡**medium intensity versus sham group; **†**high intensity versus sham group.

During the impact loading regime, an increasing trend was noticed for control and sham groups compared with the loading groups (Fig. 4 *A*). The HI group had significantly (*p* < 0.05) less BW compared with the sham group at the 10th, 11^th^, and 14th week of age (17%, 15%, and 12%, respectively; Fig. 4 *A*). The MI group had significantly less BW (13%) compared with shams at the 11th week of age only (Fig. 4 *A*). FI was also reduced for the HI and MI groups during the study period. The HI group showed reduced caloric intake compared with shams by 18%, 20%, and 17% for weeks 10, 11, and 14, respectively (Fig. 4 *B*). The MI group had reduced caloric intake by 16% and 13% for weeks 14 and 17, respectively (Fig. 4 *B*). However, no significant differences were found among the three loaded groups in terms of BW and FI during the study period. A time effect (increase in weight gain and food consumption) was observed in rats (Fig. 4 *A*, *B*). A group effect was also noticed, but no effects of group/time interaction were found. Moreover, a significant difference in FI relative to BW was observed between HI and sham groups for weeks 10, 11, and 17 during the experiment (Fig. 4 *C*).

### Long‐term effects of loading during adolescence on trabecular bone architecture

**Figure 5:**
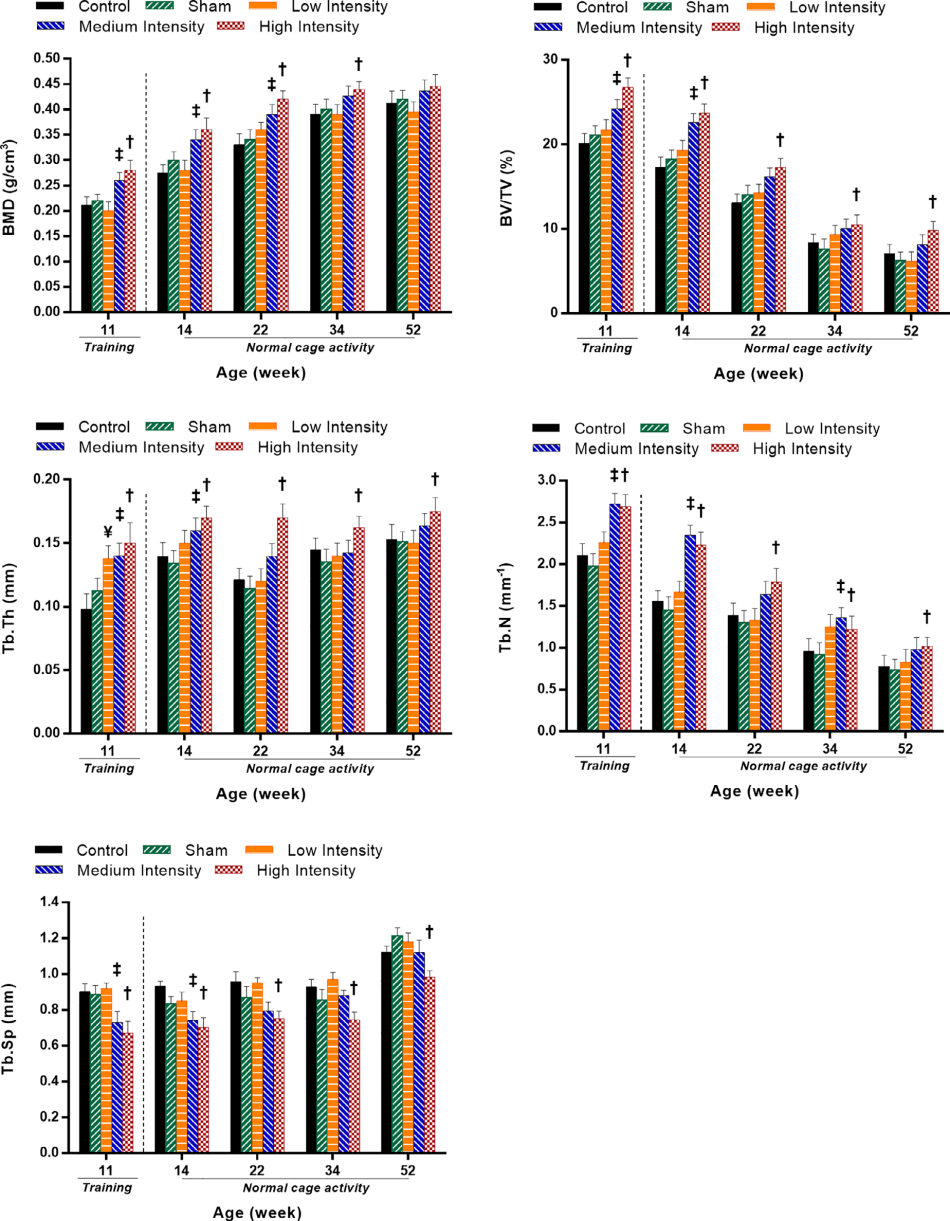
Trabecular bone morphometric parameters (means and SDs) for the five experimental groups at the end of training (11 weeks of age) and at selected normal cage activity time points (14, 22, 34, and 52 weeks of age). *p* < 0.05; **¥**low intensity versus sham group; **‡**medium intensity versus sham group; **†**high intensity versus sham group. BV/TV = bone volume fraction; Tb.N = trabecular number; Tb.Th = trabecular thickness; Tb.Sp = trabecular spacing.

The long‐term effects of pubertal loading on trabecular bone architecture were assessed by evaluating the bone structural parameters at the end of the training period (week 11) and at four intermittent times during the normal cage activity period (weeks 14, 22, 34, and 52). For each time point, HI‐loaded tibias showed significantly greater BV/TV, Tb.Th, Tb.N, and smaller Tb.Sp compared with the sham group (Fig. 5). The HI group also had higher BMD compared with the sham group, but only until the 34th week of age (Fig. 5). The MI group showed significant differences with respect to shams for BV/TV, Tb.Th, and Tb.Sp at weeks 11 and 14 (Fig. 5). There was also higher BMD for the MI group observed at the 11th, 14th, and 22nd weeks. Moreover, the MI group resulted in higher Tb.N in weeks 11, 14, and 34 (Fig. 5). No significant differences were found between the control and sham groups and among the loading groups at any time points.

### Long‐term effects of loading during adolescence on cortical bone architecture

**Figure 6:**
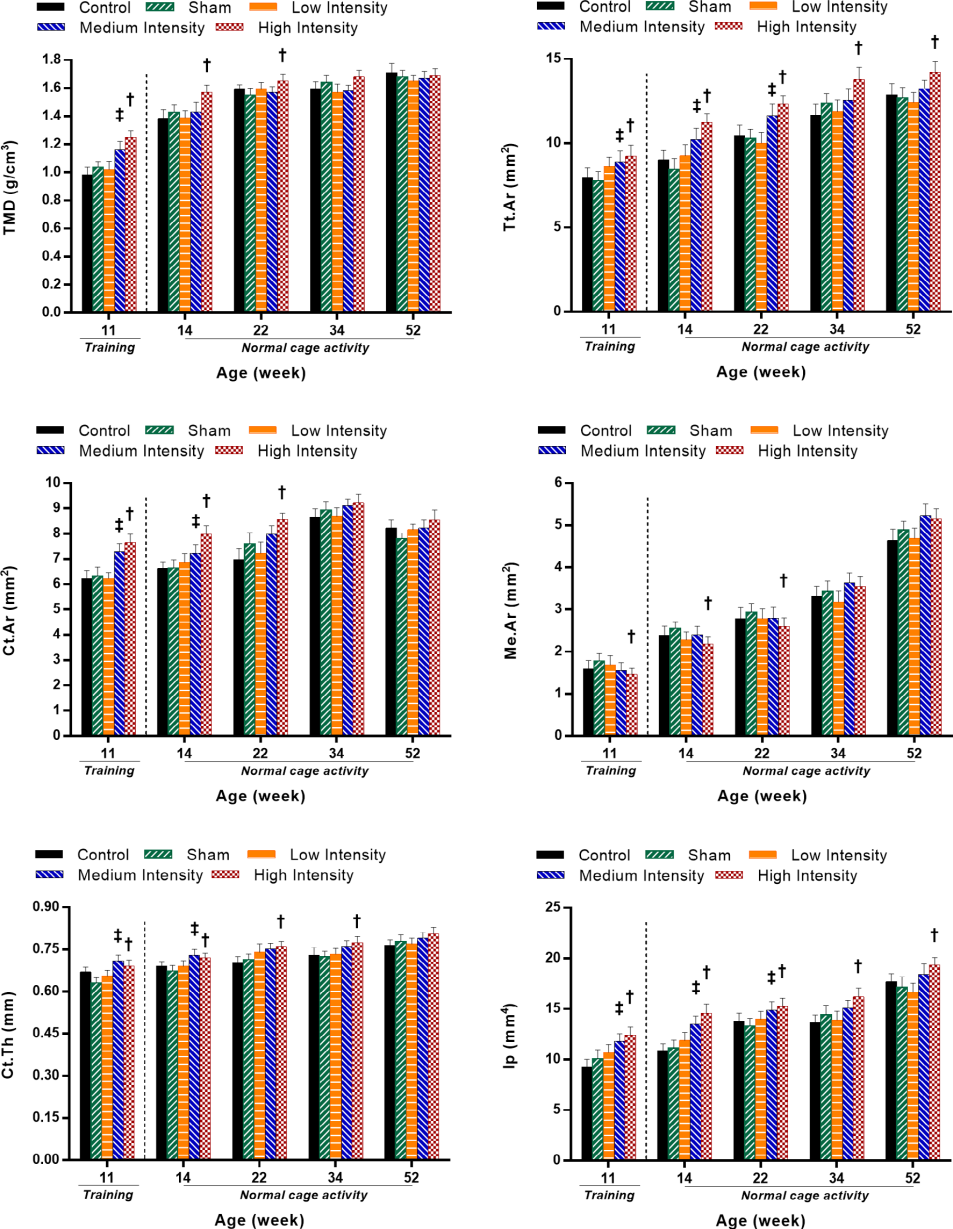
Cortical bone morphometric parameters (means and SDs) for the five experimental groups at the end of training (11 week of age) and at selected normal cage activity time points (14, 22, 34, and 52 weeks of age). *p* < 0.05; **‡**medium intensity versus sham group; **†**high intensity versus sham group. TMD = tissue mineral density; Tt.Ar = cross‐sectional area inside the periosteal envelope; Ct.Ar = cortical bone area; Ct.Th = cortical thickness.

Cortical bone structural parameters were evaluated for all rat groups at the end of the training period, as well as during the normal cage activity period to assess the effect of loading on cortical bone architecture. For each time point, loaded tibias in the HI group showed significantly greater Tt.Ar and Ip compared with the sham group (Fig. 6). The HI group also resulted in higher TMD and Ct.Ar persisting until the 22nd week, and a higher Ct.Th persisting until the 34th week compared with the sham group (Fig. 6). Also, the HI group had lower Me.Ar compared with the sham group persisting until week 22 (Fig. 6). However, the MI group showed a significant difference compared with the sham group for TMD only at the 11th week of age, for Ct.Ar and Ct.Th up to the 14th week of age, and for Tt.Ar and Ip up to the 22nd week of age (Fig. 6). No significant differences were noticed among the loading groups or between the control and sham groups during the study period.

### Muscle weight

The effect of isolated cyclic loading on muscle weight is provided in Table 1, where weights of four different muscles associated with tibia were measured for all rat groups after euthanization. Soleus muscle showed a significant weight increase in the HI (27%) and MI (16%) groups compared with the sham group. Quadriceps muscle only showed significant weight increase in the HI (12%) group compared with the sham group. However, no effect of cyclic loading was observed on gastrocnemius and tibialis anterior muscles in the loaded rats (Table 1).

### Mechanical properties of tibia

Structural and tissue‐level mechanical properties obtained from the three‐point bending tests are reported in Table 2. For structural mechanical properties, the HI group showed greater ultimate force, stiffness, postyield displacement (PYD), and postyield energy (PYE) compared with the sham group (Table 2). The MI group showed significantly higher values for only ultimate force compared with the sham group (Table 2). For estimated intrinsic mechanical properties, the HI group resulted in higher ultimate stress and toughness, whereas the MI group had only higher ultimate stress compared with the sham group (Table 2). No differences in structural or intrinsic mechanical properties were observed between the control and sham groups and among the three loading groups for the applied loading regime.

**Table 2:** Structural and Intrinsic Mechanical Properties of the Right Tibias for Control, Sham, LI, MI, and HI Groups Derived From Three‐Point Bending Tests of the Mid‐Diaphysis

Parameters/groups	Control	Sham	LI	MI	HI
Structural mechanical properties					
Yield force, F_y_ (*N*)	92.6 ± 13.8	96.3 ± 11.6	109 ± 12.7	103 ± 9.23	115 ± 12.5
Ultimate force, F_ult_ (*N*)*	138 ± 10.5	135 ± 12.7	142 ± 15.5	**156 ± 12.5** ^**α**^	**164 ± 13.7** ^**α**^
Stiffness, k (*N*/mm)*	254 ± 19.2	263 ± 17.6	282 ± 25.8	290 ± 17.6	**320 ± 25.2** ^**α**^
Postyield displacement (μm)*	0.59 ± 0.15	0.52 ± 0.11	0.57 ± 0.17	0.61 ± 0.13	**0.73 ± 0.11**
Work to yield (mJ)	19.7 ± 2.89	18.8 ± 2.61	18.2 ± 2.72	19.3 ± 2.69	20.9 ± 2.77
Postyield energy (mJ)*	82.2 ± 7.12	79.3 ± 8.24	85.2 ± 8.23	91.2 ± 9.23	**98.6 ± 7.82** ^**α**^
Work to failure (mJ)	98.1 ± 10.3	106 ± 11.3	110 ± 12.6	101 ± 10.1	118 ± 9.45
Estimated tissue‐level mechanical properties					
Yield stress, σ_y_ (MPa)	212 ± 25.4	222 ± 23.2	245 ± 26.1	232 ± 27.1	248 ± 24.1
Ultimate stress, σ_ult_ (MPa)*	310 ± 23.7	297 ± 25.1	314 ± 19.3	**339 ± 20.2** ^**α**^	**352 ± 21.1** ^**α**^
Young's modulus, E (GPa)	26.2 ± 3.56	25.2 ± 2.56	27.3 ± 3.55	25.1 ± 2.66	28.6 ± 3.41
Strain to yield (με)	8724 ± 1231	9014 ± 1244	8965 ± 989	9230 ± 1311	8655 ± 1277
Strain to failure (με)	10827 ± 1061	10566 ± 1237	10218 ± 1444	11302 ± 1023	11804 ± 1151
Resilience (MPa)	0.73 ± 0.19	0.82 ± 0.23	0.81 ± 0.17	0.78 ± 0.28	1.07 ± 0.35
Toughness (MPa)*	4.92 ± 1.07	4.63 ± 1.05	5.19 ± 1.55	5.73 ± 1.19	**7.34 ± 1.16** ^**α**^

Values are expressed as mean ± SD, *N* = 6/group for control and sham groups; *N* = 9/group for LI and HI; and *N* = 8 for MI. In the parameter column, * indicates a significant effect (*p* < 0.05) from a two‐way repeated‐measure ANOVA with Tukey's multiple comparisons test. When there was a significant effect, Tukey's post hoc pairwise comparisons evaluated whether the sham group was significantly different compared with the others. A bold value and “α” indicate a significant difference versus sham group.

LI = Low intensity; MI = medium intensity; HI = high intensity.

### Finite element analysis of tibia

**Figure 7:**
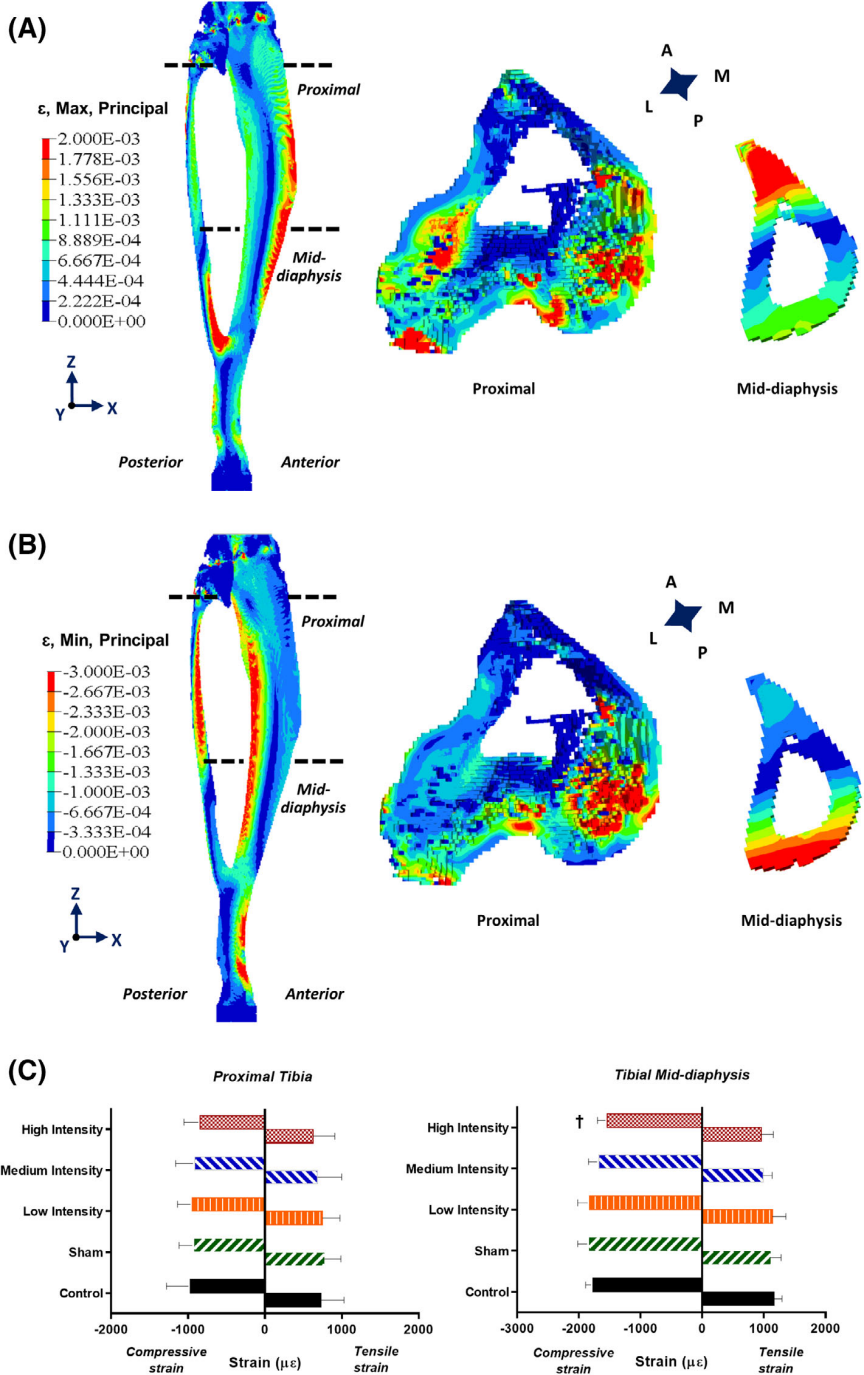
(*A*) Principal tensile strain distribution within a representative 52‐week‐old rat tibia and within corresponding transverse sections of proximal trabecular and mid‐diaphysis cortical bone volumes of interest (VOIs). (*B*) Principal compressive strain distribution within a representative 52‐week‐old rat tibia and within corresponding transverse sections of proximal trabecular and mid‐diaphysis cortical bone VOIs. (*C*) Principal compressive and tensile strains within the 52‐week‐old rat tibial proximal trabecular VOIs and mid‐diaphysis cortical VOIs for the five experimental groups. *p* < 0.05; **†**high intensity versus sham group.

Principal compressive and tensile strain distributions were evaluated for VOIs of the proximal tibia and mid‐diaphysis. For the trabecular VOI (proximal region), average principal tensile strains ranged from 627 με (±SD: 283) for the HI group to 774 με (±SD: 216) for the sham group (Fig. 7 *C*), whereas average principal compressive strains varied from 842 με (±SD: 210) for the HI group to 971 με (±SD: 311) for the control group (Fig. 7 *C*). Higher average strains were predicted in the cortical bone VOI (mid‐diaphysis). Average principal tensile strains ranged from 959 με (±SD: 194) for the HI group to 1167 με (±SD: 126) for the control group (Fig. 7 *C*), and average principal compressive strains varied between 1537 με (±SD: 162) for the HI group and 1835 με (±SD: 183) for the sham group (Fig. 7 *C*). A significant difference was observed between the HI and sham groups (Fig. 7 *C*) for the average principal compressive strains at the cortical mid‐diaphysis.

## Discussion

The results of this study suggest that performing high‐intensity cyclic loading during adolescence results in a significant advantage in terms of both trabecular and cortical bone microstructural properties. Our findings demonstrate the importance of investigating isolated cyclic loading effects during the bone growth period to elucidate the long‐term maintenance of loading‐induced bone benefits using a rodent model.

### Cyclic loading temporarily reduced body weight and food intake at the rat puberty/adult transition period

Loaded and nonloaded rats responded differently to pubertal cyclic loading with respect to BWs (BW) and FIs (FI). However, the effects incurred through adolescence only sustained around the end of the adolescence and beginning of young adulthood phases, but disappeared at adulthood. The HI group had lower BW and reduced FI compared with the sham group at 10, 11, and 14 weeks of age (Fig. 4 *A*, *B*), whereas the MI group showed lower BW only at 11 weeks of age (Fig. 4 *A*), and reduced FI at 14 and 17 weeks of age (Fig. 4 *B*), compared with the sham group. Loading is generally correlated with an increase in FI in rats.^55^, ^56^ Our results for the MI and HI groups showed the contrary, with a reduced BW coupled with a significant caloric reduction. The reduced BW could be related to the intensity of the applied tibial loading. The increased stress levels in MI and HI groups^55^ might be associated with amplified hormone secretion,^57^, ^58^ which might have triggered the observed reduction in BW.^59^ This could also be an indication of increased lean tissue in the loaded animals, where the trained rats might have used their caloric intake in the synthesis of lean tissue, rather than storing them in adipose tissue.^60^ Moreover, previous studies have shown that male rats undergoing a forced loading regime do not tend to compensate for the excessive energy expenditure with increased FI, unlike their female counterparts.^59^ Our observations are supported by previous studies, which have reported a decreased BW simultaneous with a reduced FI in adult rats after the end of forced swimming^61^ and running regimes.^55^

During the normal cage activity period, the effects from the MI and HI groups on FI disappeared after 17 and 14 weeks of age, respectively (Fig. 4 *B*). Hence, no effects remained in the long‐term. Moreover, when caloric intake was expressed relative to BW, the FI of the HI group was significantly elevated at the 10th and 11th week, and lowered at the 17th week of age compared with the sham group. However, no significant effect was observed afterward (Fig. 4 *C*). This phenomenon shows different observations between absolute and relative measurements. The MI and HI groups exhibited a reduction in absolute measurements of BW and FI, but when the FI was expressed relative to the BW, the outcome was reversed (Fig. 4 *C*). However, increments in BW in the HI and MI groups during late normal cage activity period can be considered as a natural phenomenon.^62^ It was indeed reported for both adult humans and rats that, once the loading regime is withdrawn, the BW starts increasing to match the natural level.^63^ Our study also demonstrated that even at the 52nd week of age (eg, 41 weeks after the cessation of pubertal loading), the HI and MI rats had significantly higher soleus muscle weight compared with the sham group (Table 1). The HI group also had a higher quadriceps muscle weight compared with the sham group at this period (Table 1). So, the MI and HI loadings might have produced sustained adaptive physiological responses in skeletal muscle weight, which could result from increased energy metabolism and resting metabolic rate (RMR).^62^ Increased energy metabolism and RMR are reported to enhance the oxygen utilization capacity of skeletal muscles,^64^ which is further associated with increased catecholamine hormones^64^ and lipoprotein lipase activity,^65^, ^66^ both regulators of fatty cells inside the body. All these changes might have reduced the energy available for fat storage. Therefore, the MI and HI groups gradually regained their BW to match the normal level after loading. Our findings are supportive of other human and rat studies,^67^, ^68^, ^69^ where BW was reported to be suppressed during loading period, but started to re‐increase after loading cessation. Another study^27^ also reported similar findings: Childhood running loading for 14 weeks of age did not have any effect on the BW of the loaded rats after 40 weeks of normal cage activity period.

### High‐intensity cyclic loading induced enhanced trabecular bone at the end of puberty, which was maintained during the adulthood normal cage activity period

Both the HI and MI groups exhibited pubertal loading‐induced changes in the trabecular bone structure even after the cessation of the loading. The HI cyclic loading of the rat tibias for 8 weeks in the adolescent period resulted in a trabecular microstructure with greater BMD, BV/TV, Tb.Th, and Tb.N, but less Tb.Sp compared with the sham group during the normal cage activity period. For the HI group, all measured trabecular morphometric parameters, except BMD, maintained induced benefits during the entire normal cage activity period up to 52 weeks of age (Fig. 5). However, the enhanced BMD was discontinued after 34 weeks of age (Fig. 5). For the MI group, the induced benefits disappeared at an earlier time point for most of the trabecular parameters. For the LI group, the only effect was observed immediately after the end of the loading period (11 weeks) for Tb.Th, but disappeared afterward (Fig. 5).

The BMD increment is a natural phenomenon during adolescence.^70^ In addition, it has been reported that strenuous activity during growth can significantly decrease tartrate‐resistant acid phosphatase levels in blood serum, leading to a significant increase in BMD in trabecular metaphysis.^71^, ^72^ Moreover, HI and MI cyclic loadings could have altered calciotropic hormones, which are responsible for promoting a positive calcium balance and lead to a significant increase in skeletal mass.^71^, ^73^, ^74^ Studies by Hagihara and colleagues,^71^ Iwamoto and colleagues,^75^ and Joo and colleagues^76^ confirm that BMD increases after treadmill exercise regimes in growing rats. Discontinuation of BMD benefits in HI group after 34 week of age could be related to BW and hormones.^77^, ^78^ As rats grow older and BW increases, a BW burden can influence the bone mineral content in tibia.^79^, ^80^ Around 60% of the BW is carried by the legs of rats in normal cage activities.^79^, ^80^ Hence, it could be possible that the increasing BW has negatively influenced the BMD of tibias in the HI group at adulthood and thus the benefits gained up to the 34th week of age eventually disappeared (Fig. 5). The absence of benefits in BMD at adulthood observed in this study is supported by others,^27^, ^81^ where the absence of load‐induced benefits were also reported for bone mineral content in the long‐term period.

The significant BV/TV increase during adolescence can be associated with the increase in BMD.^82^ In the normal cage activity period, BV/TV gradually decreased for all rat groups. But the HI group maintained a greater BV/TV compared with the sham group during the normal cage activity period. HI cyclic loading may have triggered an effect on the osteoclasts of trabecular bone structure, which eventually led to the inhibition of bone resorption instead of the promotion of bone formation with aging.^83^ Indeed, it has been shown that exercised animals have beneficial effects on BV/TV compared with nonexercised animals.^71^, ^84^ A significant change in Tb.Th is an indication of loading‐induced positive effects on normal bone‐growing phenomena,^85^ and the change in Tb.N is directly associated with the change in BV/TV and Tb.Th.^86^ This explains why the MI group showed discontinued positive effects on Tb.N after 14 weeks of age, simultaneously with BV/TV and Tb.Th (Fig. 5). Tb.Sp is measured by the diameter of the largest sphere that fits within the marrow space in‐between trabeculas. So, a significant decrease in Tb.Sp for the HI group compared with the sham group can be associated with the induced bone gain (increasing BMD) and gradual thickening of trabeculas (increasing Tb.Th; Fig. 5) through increased connectivity as observed in our study. Our findings for BV/TV, Tb.Sp, Tb.Th, and Tb.N are in agreement with other studies,^84^, ^85^, ^87^, ^88^, ^89^ where similar patterns also were observed for loaded and normal bone growing process for aged rats and mice bone structure.

### High‐intensity cyclic loading induced positive changes in cortical bone microstructure at the end of puberty, which partly remained during the adulthood normal cage activity period

Our findings showed that HI cyclic loading positively affected cortical bone tissue in the long‐term normal cage activity period, and to some extent for the MI cyclic loading group. The HI group maintained the loading‐induced benefits with greater total bone area and moment of inertia at the mid‐diaphysis compared with the sham group up to the end of the normal cage activity period (Fig. 6). Moreover, HI cyclic loading enhanced bone mechanical properties with increased tibial strength and toughness at the 52nd week of age (Table 2).

TMD significantly increased for the HI and MI groups at the end of loading period (Fig. 6), until the 22nd week of age for the HI group, whereas the MI group lost this benefit after the training period. The loss of TMD benefits during the normal cage activity period could be related to porous structural modification with rat aging process. Indeed, BMD can be affected by the induction of new cortical pores and not simply by the enlargement of the diameter of existing cortical pores with increasing age.^90^, ^91^ These counteracting effects in cortical pore generation with age may be responsible for the observed reduced TMD in the HI group during the normal cage activity period. Me.Ar, which represents the area enclosed by endocortical perimeter, showed an increasing pattern with age for all groups of rats (Fig. 6). Ct.Ar represents the area between periosteal and endocortical surfaces. Hence, a significantly lower Me.Ar in the HI group compared with the sham group might be correlated to the increased Ct.Ar for the HI group up to the 22‐week period (Fig. 6). Increased Ct.Ar is also associated with the reduced strain distribution on the bone surface.^92^ Hence, the reduced (19.3%) average principal compressive strain in the HI group compared with the sham group from our FE analyses can be justified (Fig. 7 *C*).

The continuous increase in radial growth (Ps.Pm; Fig. SS1) for the HI group, along with an increase in the Ct.Th, eventually led to an increase in total bone area, which remained consistent during the entire normal cage activity period. Our findings are supported by another study,^93^ where impact loading (jumping) was reported to increase cortical bone area primarily because of an increase in the periosteal perimeter, with little changes in the endocortical perimeter or the medullary area. The exact reason why Ps.Pm remained significantly higher in the HI group, even after keeping both Ec.Pm (Fig. SS1) and Me.Ar (Fig. 6) unchanged, is not apparent. It might result from a high impact influencing the bony structure by redistributing the bony materials from the endosteal region toward the periosteal region.^93^, ^94^ This phenomena also triggered a shift in the mass distribution with respect to the bone neutral axis, which significantly increased the polar area moment of inertia (Ip)^95^, ^96^ in the HI cyclic group (Fig. 6).

The HI cyclic group had greater ultimate force, stiffness, PYD, PYE, ultimate stress, and toughness compared with the sham group (Table 2), whereas the MI cyclic group only exhibited significant increases in ultimate force and stress (Table 2). Having higher ultimate force and ultimate stress indicate that the loaded tibias in the MI and HI groups at adulthood can sustain greater load before fracture compared with the LI and nonloaded tibias. The total bone area is reported to be a key factor in determining the ultimate force^97^, ^98^; it was shown to increase in the HI cyclic group. Ultimate stress (σ_ult_) can be directly correlated to the ultimate force (F_ult_) experienced by the bony samples,^44^, ^99^ so a significant increase in both σ_ult_ and F_ult_ for the MI and HI groups can be explained (Table 2). Bone stiffness is associated with Tt.Ar, Ct.Th, and Ct.Ar for any given sample^100^, ^101^; therefore, the increase in bone stiffness for the HI group can be explained (Table 2). Overall, the enhanced morphometric parameters for the HI and MI groups during the normal cage activity period and at adulthood support the consequently improved mechanical properties. PYD, which is a measure of ductility,^102^ and PYE significantly increased in the HI group compared with the sham group. The greater PYD can be correlated to the higher stiffness and failure strength observed in the HI group.^100^, ^101^, ^103^ Bone toughness, which represents a measure of resistance to fracture, depends primarily on work to failure and bone width in the anteroposterior direction.^104^ The enhanced toughness of the HI cyclic group at adulthood could hence partly result from the increased Ct.Th and Ps.Pm.

Our results disagree with some published studies where increased bone mass and enhanced bone geometry were reported to disappear after 4 and 28 weeks of a normal cage activity period, respectively, in growing rats experiencing treadmill running exercise for 8 weeks^19^ and 14 weeks.^105^ Potential explanations for these dissimilarities might include the type of loadings used (tibial compression versus treadmill running), site of investigation (tibial proximal metaphysis and mid‐diaphysis versus to femoral neck and midshaft), induced strain level (controlled calibrated strain versus uncontrolled strain), and overall experimental study design (short detraining period versus long detraining period). Also, the use of different imaging techniques with a different scanner setup for bone morphological properties (μCT versus pQCT) might have contributed to the observed differences. However, the observed enhanced mechanical properties for the MI and HI cyclic groups in this study agree with several previous findings. In a rodent study,^84^ where 16‐week‐old mice tibia were loaded for 4 weeks, improved bone morphology, along with enhanced postyield properties, was reported after 52 weeks of a normal cage activity period. In separate human studies, enhanced bone properties were maintained in the primarily used arm after 5 years of detraining for female tennis players^106^ and for a lifetime period for professional baseball players.^3^, ^107^

### Strengths and limitations

The present study has some strengths over published studies, namely the refined longitudinal investigation of both trabecular and cortical morphometric properties. Changes in bone tissue properties were tracked after the loading period (11 weeks of age) until rats reached 1‐year old. This follow‐up period (up to 41 weeks) provided enough time to observe bone adaptation phenomena induced by a pubertal loading regime and was refined enough to assess the time point at which bone morphological changes occurred or disappeared. The use of a sham group of rats also represents a strength of the completed study; it allowed isolating the effects of cyclic loading when comparing shams with LI, MI, or HI rats, whereas the comparison of shams with controls isolated the effects of rat manipulation and handling. Also, our cyclic loading conditions were precalibrated with strain gauge measurements using rat tibias of different age groups. These measurements were validated numerically using a finite element modeling tool described in a previous study.^49^ This methodological approach allowed us to apply finely controlled loading with known resulting strain conditions in the tibia.

The present study also includes some limitations. A relatively low number of rats was used for the control and sham groups, although the sample‐size calculation was adapted from previous studies. Data from the literature showed that six rats per group is a minimum number to obtain statistically significant differences in bone morphological parameters among the groups.^108^, ^109^ Hence, our control and sham groups consisted of six animals, whereas each impact loading group consisted of 10 animals. The loading conditions used in this study varied not only in terms of displacement (strain) magnitude, but also in terms of the acceleration applied during loading and unloading. This acceleration and deceleration phenomena were not reported in this study. Only strains encountered in the anteromedial location of the tibia were reported. In a previous study,^49^ it was observed that maximum strains can occur at the posterolateral regions at the same tibial level. As our calibration was performed for the anteromedial tibial location, for improved strain gauge installation, we chose this site for our primary investigation. Also, a decline in the BV/TV value in our study despite the increase in BMD might be associated with the contribution from the cortical shell area during the segmentation process. The bone segmentation algorithm used in the current study would need to be modified and refined better to solve this problem. The use of a rat model for long‐term study has drawbacks. Rats have been reported to possess a limited ability for cortical bone resorption during a detraining period because of a lack of secondary remodeling of Haversian canals^110^ and to continue their growth until relatively late in life. However, a previous study reported the existence of bone remodeling in the cortical bone structure of adult rats in response to mechanical stimuli.^111^ Hence, the use of rat models for investigating long‐term effects of mechanical stimuli on bone microstructure was considered adequate for the objectives of this study.

## Conclusion

In summary, our data indicated that adolescent cyclic loading prompted a strong anabolic response in both trabecular and cortical bone microstructure at the end of growth and that it was maintained up to the 52nd week of age in male rats. BWs and FIs in the MI and HI groups were decreased during the transition period of adolescence and young adulthood phase, but these effects disappeared at adulthood. HI cyclic loading allowed maintaining improved trabecular microstructure along with enhanced cortical bone size and improved strength at adulthood. Overall, our findings suggest that even though both trabecular and cortical bone drifted through age‐related changes during rat aging, HI cyclic loading performed during adolescence can preserve the benefits in bone microstructure and strength for at least 41 weeks following a training period.

## Disclosures

The authors declare no competing interests.

## Supporting information


**Fig S1**:Mean values and standard deviations of the periosteal and endocortical perimeter for the five experimental groups at the end of training (11 week of age) and at selected detraining time points (14, 22, 34, and 52 weeks of age)

**Table SB1**:ANOVA test with Tukey's multiple comparisons for the lengths of tibias (mm) from control, sham, LI, MI and HI groups of rats extracted at the end of experiment.
