# Supplementary material for: Isolated Cyclic Loading During Adolescence Improves Tibial Bone Microstructure and Strength at Adulthood
Source: JBMR Plus. 2020 Mar 11;4(4):e10349. doi: 10.1002/jbm4.10349 (PMC7117850; doi:10.1002/jbm4.10349)

Supplemental Figure 1: Mean values and standard deviations of the periosteal and endocortical perimeter for the five experimental groups at the end of training (11 week of age) and at selected detraining time points (14, 22, 34, and 52 week of age)

$p < 0.05$ ‡medium impact vs. sham; †high impact vs. sham

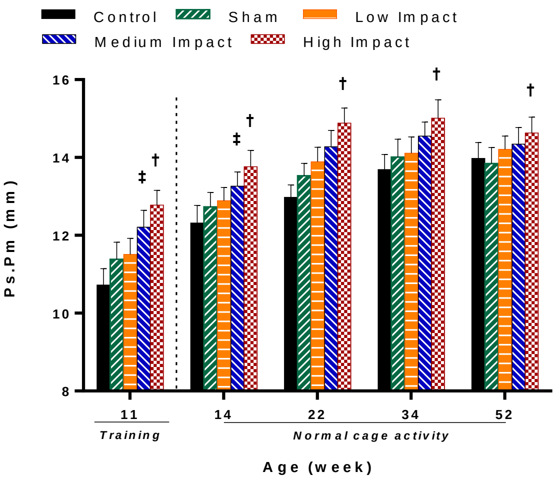

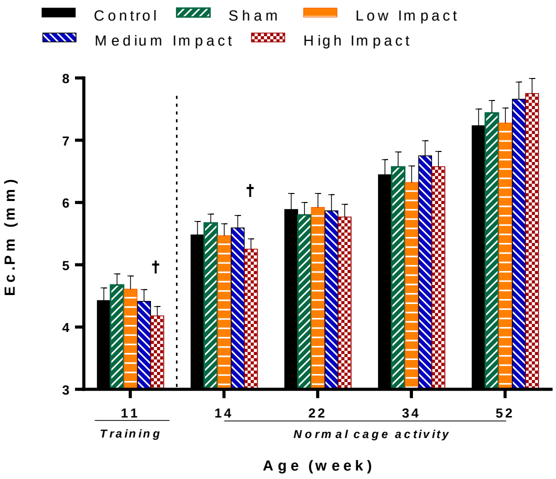

Supplement: Supplementary file 1 — Fig S1:Mean values and standard deviations of the periosteal and endocortical perimeter for the five experimental groups at the end of training (11 week of age) and at selected detraining time points (14, 22, 34, and 52 weeks of age) [file JBM4-4-e10349-s001.pdf]
