# Supplementary material for: Isolated Cyclic Loading During Adolescence Improves Tibial Bone Microstructure and Strength at Adulthood
Source: JBMR Plus. 2020 Mar 11;4(4):e10349. doi: 10.1002/jbm4.10349 (PMC7117850; doi:10.1002/jbm4.10349)
Supplement: Supplementary file 2 — Table SB1:ANOVA test with Tukey's multiple comparisons for the lengths of tibias (mm) from control, sham, LI, MI and HI groups of rats extracted at the end of experiment. [file JBM4-4-e10349-s002.docx]

**Table B1**: ANOVA test with Tukey’s multiple comparisons for the lengths of tibias (mm) from control, sham, LI, MI and HI groups of rats extracted at the end of experiment.

| *Groups* | Tibiae length (mm) |
| --- | --- |
| Control | 51.8 ± 1.01 |
| Sham | 52.2 ± 0.72 |
| Low Impact (LI) | 50.5 ± 0.65 |
| Medium Impact (MI) | 49.8 ± 0.73 |
| High Impact (HI) | 48.9 ± 1.10 |

Values are expressed as Mean ± SD, n = 6/group for control and sham; n = 10/group for LI, MI and HI.
